# Acromegaly and hyperparthyroidism: about a rare association

**DOI:** 10.11604/pamj.2018.29.12.14404

**Published:** 2018-01-04

**Authors:** Mohamed Chermiti, Dhia Kaffel

**Affiliations:** 1 Nephrology Department, Kef Hospital, Tunisia; 2 Rheumatology Department, Kassab Institute, Manouba, Tunisia

**Keywords:** Hyperparthyroidism, acromegaly, pituitary macroadenomas

## Image in medicine

Here we report a case of 49-year-old Tunisian man followed at our consultation for bone pain. He complained of enlargement of the hands and feet since the age of 23. End stage renal failure secondary to hypertension and diabetes was discovered at the age 39. He was also operated at this age for a synchronous GH- and prolactin-secreting pituitary macroadenomas. The examination shows a frontal boss associated with macroglossia. In biology: normocalcemia at 2.16 mmol/l (normal range, 2.1-2.6 mmol/l), normophosphatemia at 1.2 mmol/l (normal range, 0.8-1.5 mmol/l) and increased alkaline phosphatase level of 4647 IU/l (normal range, 42-160 IU/l). Parathyroid hormone was increased (2788 pmol/l (normal range 100-300 μg/ml in hemodialysis patients)) as well as growth hormone (122 ng/ml (normal range, < 5 ng/ml)). 25-OH vitamin D was normal (32 ng/ml (normal range, 30-70 ng/ml)). X-rays of the hands (A) and the lateral X-ray of the skull (B) combine images of hyperparathyroidism and acromegaly. An MRI of the brain found after administration of gadolinium a recurrence of the pituitary macroadenoma. Cervical ultrasound showed a single parathyroid adenoma. The patient was referred to neurosurgeons and head and neck surgeons.

**Figure 1:**
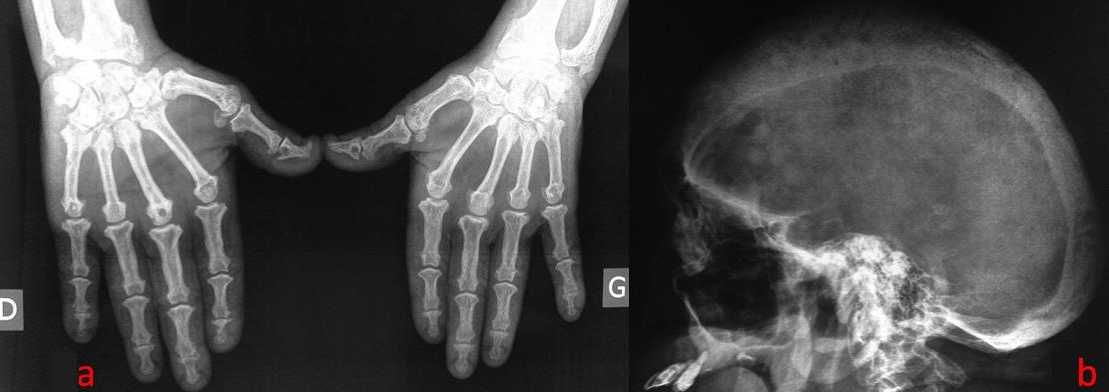
Association of radiological signs of acromegaly and hyperparathyroidism: (A) hands radiography: acromegaly: widening of the bases of distal phalanges, some hypertrophied terminal phalangeal tufts (spade appearance), some joint spaces minimally enlarged and premature osteoarthritis; hyperparathyroidism: triangular fibrocartilage complex chondrocalcinosis, acro-osteolysis of the 5th left phalangeal tufts and subperiosteal bone resorption; (B) lateral radiograph of skull: acromegaly: enlarged sella turcica with thickened skull vault and pneumosinus dilatans; hyperparathyroidism: pepper pot skull (salt and pepper sign)

